# Association of CT HU Values with Adjacent Vertebral Fractures After Balloon Kyphoplasty

**DOI:** 10.3390/medicina61091517

**Published:** 2025-08-23

**Authors:** Hiromitsu Takano, Hidetoshi Nojiri, Shota Tamagawa, Arihisa Shimura, Juri Teramoto, Hisashi Ishibashi, Yuta Sugawara, Kazuki Nakai, Muneaki Ishijima

**Affiliations:** 1 Department of Orthopedic Surgery, Juntendo University, Tokyo 113-8421, Japan; hnojiri@juntendo.ac.jp (H.N.); s-tamagawa@juntendo.ac.jp (S.T.); a-shimura@juntendo.ac.jp (A.S.); jooka@juntendo.ac.jp (J.T.); h-ishibashi@juntendo.ac.jp (H.I.); y.sugawara.ke@juntendo.ac.jp (Y.S.); k.nakai.qj@juntendo.ac.jp (K.N.); ishijima@juntendo.ac.jp (M.I.); 2 Spine and Spinal Cord Center, Juntendo University Hospital, Tokyo 113-8421, Japan

**Keywords:** osteoporotic vertebral fracture (OVF), balloon kyphoplasty (BKP), adjacent vertebral fracture (AVF), Hounsfield units values

## Abstract

*Background and Objectives*: Although adjacent vertebral fractures (AVF) frequently occur after balloon kyphoplasty (BKP), their risk factors remain unclear. This retrospective study aimed to identify risk factors for AVF and evaluate the utility of Hounsfield unit (HU) values on preoperative vertebral computed tomography (CT) scans as predictors of its occurrence. *Materials and Methods*: We retrospectively evaluated 180 patients (46 male and 134 female individuals; mean age: 80.3 years; range: 60–94 years) who underwent BKP for osteoporotic vertebral fractures (OVFs) between 2021 and 2023 with at least 6 months of follow-up. The patients were categorized into the AVF (*n* = 31) and non-AVF (*n* = 149) groups. Analyzed variables included patient characteristics, fracture level, prior fractures, posterior wall injury, intravertebral cleft, vacuum phenomenon in adjacent intervertebral discs, injury-to-surgery interval, cement volume, kyphosis angles, wedge ratios, and HU values. HU values were measured at three levels on preoperative CT scans in the vertebrae above and below the treated segment. Cutoff HU values predictive of AVF were determined using receiver operating characteristic (ROC) curve analysis. *Results*: AVF incidence was 17.2% (31/180), with 71.0% occurring in the vertebrae above the treated level. HU values in all measured slices were significantly lower in the AVF group. The mean HU values in the upper vertebra were 61.1 ± 6.03 (AVF) and 84.7 ± 2.75 (non-AVF), and in the lower vertebra, 51.5 ± 8.44 and 81.0 ± 3.85, respectively. ROC analysis showed cutoff HU values of 79.3 and 61.0 for the upper and lower vertebrae, respectively. HU values were identified as independent AVF risk factors. *Conclusions*: Preoperative vertebral HU values are independent AVF predictors. Values below 79.3 in the upper or 61.0 in the lower vertebrae were linked to higher AVF risk, suggesting HU measurement is a simple, useful tool for preoperative risk assessment.

## 1. Introduction

Japan is currently a super-aged society, with an estimated 12.8 million people affected by osteoporosis (3 million men and 9.8 million women). The prevalence of osteoporosis among women in their 70s is approximately 30% [1]. Consequently, osteoporotic vertebral fractures (OVF) are becoming increasingly common. Balloon kyphoplasty (BKP) and percutaneous vertebroplasty (PVP) are minimally invasive surgical techniques used to treat OVFs. In Japan, BKP has been covered by public health insurance since 2011, leading to its widespread adoption for treating OVF. BKP is commonly used to achieve rapid pain relief; promote early ambulation and return to daily activities; prevent further collapse of fractured vertebrae; and maintain spinal alignment. It improves patients’ quality of life and has demonstrated clinical utility [2,3,4]. However, adjacent vertebral fractures (AVFs) are significant complications of BKP [5,6,7]. AVFs may adversely affect clinical outcomes; their occurrence has traditionally been difficult to predict using standard imaging methods [8]. Recent studies have highlighted the utility of computed tomography (CT) Hounsfield unit (HU) values as a quantitative indicator of bone mineral density (BMD) and as a useful tool for assessing osteoporosis and fracture risk [3]. In particular, HU values measured from vertebral trabecular bone have shown strong correlations with BMD. This approach is gaining attention due to its simplicity and accessibility [9,10]. Although many reports have documented the occurrence of AVFs after BKP, the associated risk factors remain poorly understood. Therefore, this study retrospectively investigated the factors associated with the occurrence of AVFs. Specifically, we focused on the utility of vertebral HU values measured on preoperative CT scans for predicting the risk of AVFs.

## 2. Materials and Methods

### 2.1. Patients

This retrospective study included 180 patients (46 men and 134 women; mean age, 80.3 years; range, 60–94 years) who underwent BKP for OVF between 2021 and 2023 at our institution or affiliated hospitals and were followed up for at least 6 months postoperatively. The patients were divided into two groups: those who developed AVFs (AVF group, *n* = 31) and those who did not (non-AVF group, *n* = 149). Patients with steroid-induced osteoporosis, pathological fractures, or diffuse idiopathic skeletal hyperostosis (DISH) were excluded. All patients wore a soft brace for 3 months postoperatively and were prescribed osteoporosis medications—such as teriparatide, romosozumab, or anti-RANKL antibodies—whenever possible.

### 2.2. Data Collection

**Figure 1:**
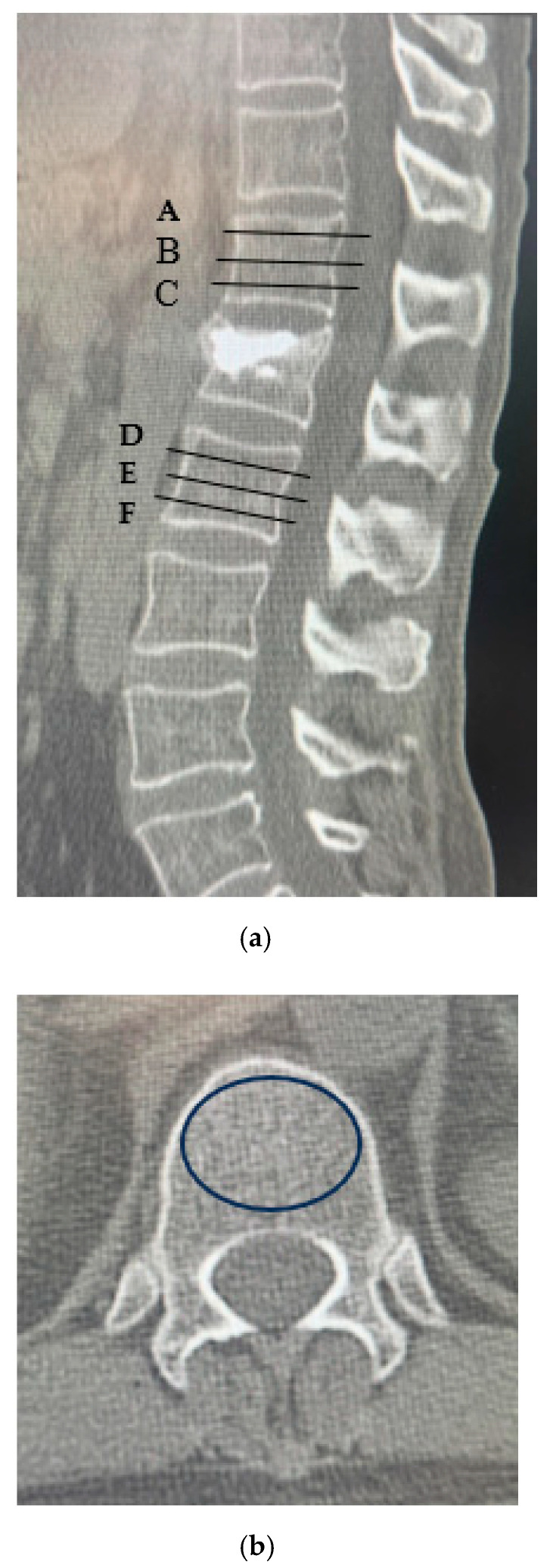
Measurement of HU values. (**a**) Computed tomography scan illustrating the method for determining HU values using an elliptical region of interest function. Slice A was placed just inferior to the superior endplate. Slice B was selected from the center of the body. Slice C was placed just superior to the inferior endplate. Slice D was placed just inferior to the superior endplate. Slice E was selected from the center of the body. Slice F was placed just superior to the inferior endplate; (**b**) For each measurement, the largest possible elliptical region of interest was drawn, excluding the cortical margins to prevent volume averaging. Abbreviation: HU, Hounsfield unit.

**Figure 2:**
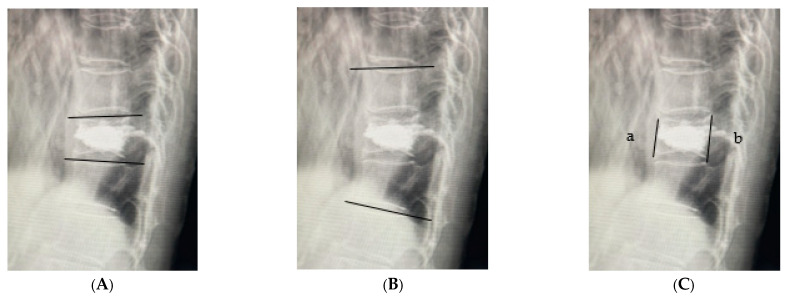
Measurement of plain lateral radiography. (**A**) Vertebral kyphosis angle; (**B**) Local kyphosis angle; (**C**) Vertebral wedge ratio (a/b × 100).

CT was performed using a 64-slice multidetector CT scanner (SOMATOM Go. Top, Siemens Healthineers, Erlangen, Germany) with standardized settings; HU values were measured. Following the method described by Schreiber et al. [9], six elliptical regions of interest (ROI) were drawn within the trabecular bone of the vertebrae one level above and below the fractured vertebra: the upper, middle, and lower third of each vertebra (labeled A to F). The mean HU values were recorded for each region (Figure 1). To evaluate the reliability of HU measurements, two independent spine surgeons assessed CT images in 30 randomly selected patients. Inter- and intra-observer agreements were assessed using intra-class correlation coefficients (ICC). The following parameters were investigated: preoperative patient characteristics, fracture level, presence of old vertebral fractures, posterior wall injury, intravertebral cleft, vacuum phenomenon in the adjacent intervertebral disc, time from injury to surgery, cement volume, occurrence of AVF, posterior vertebral kyphosis angle of the affected vertebra, vertebral wedge ratio, and local kyphotic angle on plain lateral radiography preoperatively, immediately postoperatively, and at the final follow-up (Figure 2). An AVF was defined as a new compression fracture at the vertebra immediately above or below the treated vertebra, confirmed by ≥20% vertebral height loss on radiographs and/or magnetic resonance imaging evidence of bone marrow edema with new-onset back pain [7,11].

### 2.3. Statistical Analysis

The normality of continuous variables was tested using the Shapiro–Wilk test. Depending on the distribution, comparisons were made using either Student’s *t*-test or Mann–Whitney U test. Categorical variables were compared using the chi-squared test. Univariate logistic regression was performed to explore potential associations with AVF. Clinically relevant variables and those reported as risk factors in the literature were fitted in the multivariate logistic regression analysis, regardless of univariate significance, to minimize selection bias and ensure robust modeling. Statistical significance was set at *p* < 0.05. Statistical analyses were performed using the JMP Pro software (version 18.0; SAS Institute, Cary, NC, USA). The cutoff HU values for predicting AVF were determined using receiver operating characteristic (ROC) curve analysis. This study was approved by the Research Ethics Committee of the Faculty of Medicine, Juntendo University (approval code: E24-0177, approved on 9 September 2024). Written informed consent was obtained from all participants.

## 3. Results

**Figure 3:**
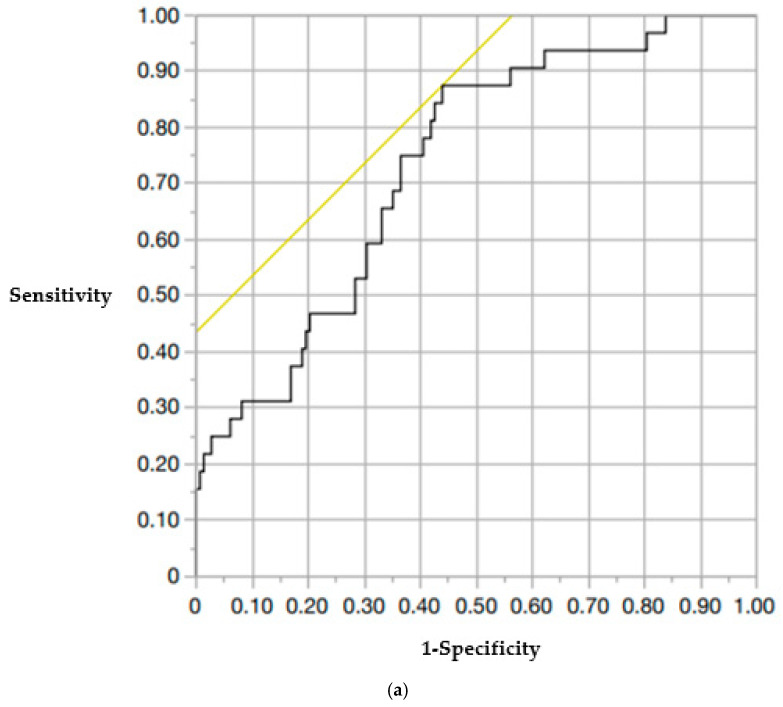

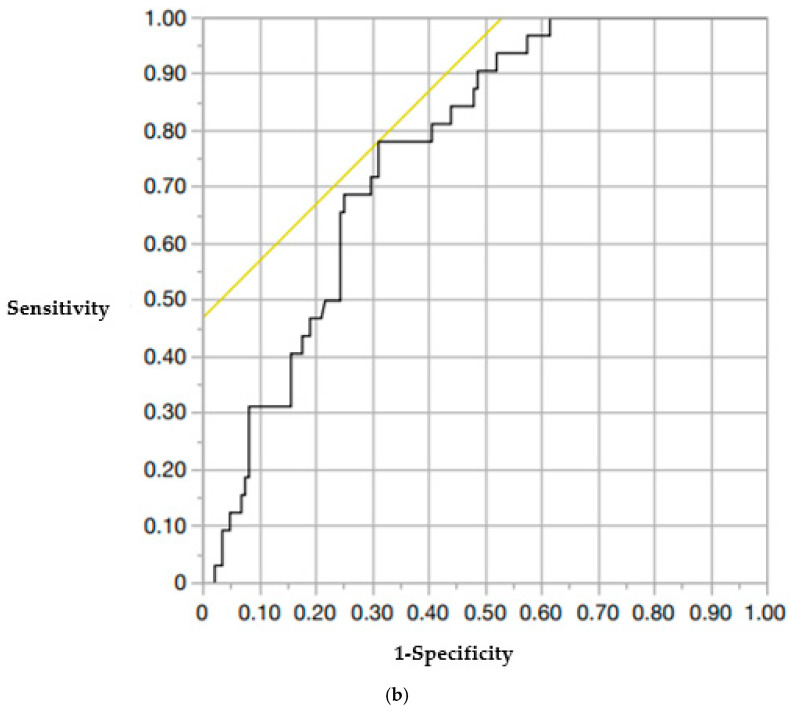
ROC curve. (**a**) ROC curve showing the cutoff HU value for the upper adjacent vertebra (cutoff, 79.3). (**b**) ROC curve showing the cutoff HU value for the lower adjacent vertebrae (cutoff 61.0).

The incidence of AVF in this study was 17.2% (31 out of 180 cases). The patients were divided into an AVF group (*n* = 31) and a non-AVF group (*n* = 149). In the AVF group, the mean age was 79.9 ± 0.6 years, while in the non-AVF group, it was 82.0 ± 1.3 years. The sex distribution was as follows: 8 male and 23 female patients in the AVF group, and 37 male and 112 female participants in the non-AVF group. No significant differences were found between these parameters. The mean young adult mean (YAM) values were 68.0 ± 2.89 in the AVF group and 66.2 ± 1.37 in the non-AVF group. Posterior wall injury was present in 48.4% (15/31) and 32.9% (49/149) of patients in the AVF and non-AVF groups, respectively. Intravertebral clefts were found in 35.5% (11/31) and 26.9% (40/149) of patients in the AVF and non-AVF groups, respectively. Vacuum phenomena in the adjacent intervertebral discs were present in 25.8% (8/31) and 11.4% (17/149) of patients in the AVF and non-AVF groups, respectively. None of these differences were statistically significant. Cement volume was the same in both groups (7.3 ± 0.27 mL vs. 7.3 ± 0.12 mL). Preoperative vertebral kyphosis angles were −10.7 ± 0.91° in the AVF group and −9.5 ± 0.41° in the non-AVF group. Immediately postoperative angles were −4.7 ± 0.59° and −4.4 ± 0.27°, and final postoperative angles were −7.9 ± 0.86° and −6.6 ± 0.39°, respectively. There were no significant differences between the groups. Preoperative local kyphosis angles were −11.3 ± 2.54° in the AVF group and −6.3 ± 1.16° in the non-AVF group. Immediately postoperative local kyphosis angles were −0.9 ± 3.05° and −1.1 ± 1.39°in the AVF and non-AVF groups, respectively. The vertebral wedge ratios immediately postoperatively were 86.5 ± 1.58% and 88.6 ± 0.72%, and at final observation, 78.7 ± 2.33% and 82.1 ± 1.05%, respectively. No significant differences were observed in these parameters, except for the final postoperative local kyphosis angle and preoperative vertebral wedge ratio, which were significantly different (Table 1). The thoracolumbar spine (T11–L2) was involved in 93.5% (29/31) of patients in the AVF group and 77.2% (115/149) of patients in the non-AVF group, with a significantly higher proportion in the AVF group (*p* < 0.05). The mean number of old vertebral fractures was also significantly higher in the AVF group (0.90 ± 0.18 vs. 0.59 ± 0.09, *p* < 0.05) (Table 1). Among AVF cases, 71.0% (22/31) occurred in the upper adjacent vertebrae (Table 2). The inter-observer reliability of HU measurements was excellent, with an ICC of 0.89. The intra-observer reliability was also excellent, with an ICC of 0.92. The mean HU values in the six ROI (A–F) were significantly lower in the AVF group than those in the non-AVF group (*p* < 0.05). Specifically, HU values in the AVF group were as follows: A: 53.6 ± 68.9, B: 57.3 ± 72.3, C: 62.9 ± 67.2, D: 50.7 ± 69.7, E: 46.8 ± 71.2, F: 47.9 ± 68.1. In the non-AVF group, the corresponding values were as follows: A: 94.0 ± 79.5, B: 76.7 ± 81.0, C: 86.2 ± 76.2, D: 94.3 ± 79.6, E: 72.2 ± 85.7, F: 83.0 ± 83.4 (Table 3). When comparing mean HU values, the upper vertebral (A–C) mean HU was 61.1 ± 6.03 in the AVF group and 84.7 ± 2.75 in the non-AVF group. The lower vertebral (D–F) mean HU was 51.5 ± 8.44 in the AVF group and 81.0 ± 3.85 in the non-AVF group. Both the upper and lower vertebral HU values were significantly lower in the AVF group (*p* < 0.05) (Table 4). The cutoff values for predicting AVF occurrence were determined by ROC curve analysis: upper vertebral HU: 79.3 (area under the curve [AUC], 0.73; sensitivity, 0.88; specificity, 0.44) and lower vertebral HU: 61.0 (AUC, 0.76; sensitivity, 0.78; specificity, 0.47) (Figure 3). Multivariate analysis identified both upper and lower vertebral HU values as independent risk factors for AVF: Upper vertebral HU: adjusted odds ratio [OR], 0.97 (95% confidence interval [CI]: 0.95–0.99, *p* = 0.043), Lower vertebral HU: adjusted OR 0.98 (95% CI: 0.96–0.99, *p* = 0.048) (Table 5).

## 4. Discussion

### 4.1. Incidence of AVF and the Importance of Early Surgery

BKP allows for early pain relief and ambulation and has been reported to reduce mortality [12]. Early intervention with BKP in patients experiencing insufficient pain relief from an OVF has been shown to improve pain, preserve activities of daily living (ADL), and prevent medical complications [13,14,15]. Although AVF is a known complication of BKP, several studies have suggested that early BKP may reduce its incidence [16]. The reported AVF rates range between 14% and 35.7% [5,6,17,18]; the rate observed in this study (17.2%) is consistent with that in previous reports. Among the AVFs observed, 71.0% occurred above treated vertebrae. The predominance of upper vertebral fractures may be attributed to biomechanical factors. BKP significantly increases the stiffness of the treated vertebrae due to cement augmentation, which alters stress distribution across adjacent vertebrae. This resulting stress concentration in the upper adjacent vertebrae is thought to increase the risk of fracture [19,20]. Although prior studies have linked high cement volumes with AVF [21], our analysis did not find a significant association. This discrepancy may be due to the relatively uniform cement volumes used in our cohort.

### 4.2. Fracture Location

In this study, OVFs in the AVF group were significantly more likely to occur at the thoracolumbar junction (T11–L2). The thoracolumbar spine lies between the rigid thoracic spine, which is stabilized by the rib cage, and the mobile lumbar spine. Therefore, it is subjected to significant mechanical stress. The anatomical and mechanical differences between these spinal regions are a major contributing factor to the high incidence of fractures in this area [22].

### 4.3. Bone Density Assessment

BMD measured using dual-energy X-ray absorptiometry (DEXA) may be overestimated in the presence of degenerative changes or aortic calcification [23]. With recent advancements in multi-slice CT technology, image reconstruction has become faster and more precise, enabling improved assessment of vertebral microarchitecture. HU values derived from CT represent physical measurements of tissue X-ray attenuation, where water is defined to 0 HU and air to −1000 HU, and are routinely used in radiology. Recent studies have reported that HU values strongly correlate with BMD and are useful for diagnosing osteoporosis [9,24]. The HU values of the vertebral trabecular bone have been shown to approximate volumetric BMD [10,25]. Furthermore, low HU values have been associated with adverse outcomes in spinal surgery, including cage subsidence after fusion [26,27], pedicle screw loosening [28,29], adjacent vertebral fractures, and poor fusion rates [9,30]. Therefore, the preoperative measurement of HU values may be a valuable tool for spinal surgery. Most studies reported HU thresholds between 120 and 130, which are indicative of bone fragility. However, no consensus has been established. In this study, the average HU values were relatively low, possibly reflecting that all included patients had OVF and thereby inherently reduced bone quality. A previous study reported that a vertebral HU threshold of 78.5 corresponds to a T-score of −2.5 SD [9]. In the context of pedicle screw loosening, an HU threshold <120 has been associated with increased risk [28]. No prior studies have evaluated HU cutoff values for predicting AVF, to the best of our knowledge. In this study, the cutoff values for predicting AVF were 79.3 and 61.0 for the upper and lower vertebrae, respectively. In our study, HU values were obtained from standard preoperative CT scans routinely used for surgical planning, without additional radiation exposure. Nonetheless, the increased radiation dose associated with CT compared with DEXA should be considered when selecting candidates for HU-based risk assessment. A limitation of this study is the absence of systematic DEXA evaluation, which precluded correlation analysis between HU values and BMD. Although HU values correlate strongly with BMD in previous studies, the lack of direct comparison might have affected the generalizability of our findings. The HU cutoff values should be interpreted in conjunction with other clinical risk factors, as they are not absolute predictors when used alone. Although the short follow-up duration limited the long-term assessment of AVF incidence and outcomes, it is noteworthy that most AVFs occurred within 2 months postoperatively [7], supporting the relevance of our findings. Other limitations included the retrospective study design, potential radiation exposure, and variability among CT machines, which might have affected HU measurements. Notably, anabolic therapy was initiated only after BKP in this study. Although this reflects current practice patterns at our institutions, preoperative administration of agents such as teriparatide or romosozumab might have altered the incidence of AVF by improving bone quality. Despite these limitations, our findings highlight the importance of preoperative HU evaluation for predicting AVF risk. Future prospective studies are warranted to validate these results.

## 5. Conclusions

In this study, we retrospectively assessed the association between vertebral HU values and AVF occurrence following BKP. The patients were divided into AVF and non-AVF groups; the mean HU values were significantly lower in the AVF group. Among all factors analyzed, HU values were identified as the only independent risk factors for AVF. Specifically, preoperative HU values of ≦79.3 in the upper vertebra and ≦61.0 in the lower vertebra were associated with an increased risk of AVF. Measuring HU using preoperative CT is a simple and practical method that may serve as a useful tool for predicting AVF risk prior to BKP. These findings suggest that HU values should be incorporated into preoperative assessments to guide treatment decisions and improve patient outcomes.

## Figures and Tables

**Table 1:** Univariable Analysis of Patient Characteristics Between AVF and Non-AVF Groups.

	AVF Group (*n* = 31)	Non-AVF Group (*n* = 149)	*p*-Value
Age (years)	79.9 ± 0.6	82.0 ± 1.3	N.S.
Sex (male/female)	M8/F23	M37/F112	N.S.
Time from injury to surgery (days)	22.9 ± 3.88	16.5 ± 1.77	N.S.
YAM (%)	68.0 ± 2.89	66.2 ± 1.37	N.S.
Fracture Level			
Thoracic spine (-T10)	0 (0%)	0 (0%)	N.S.
Thoracolumbar spine (T11-L2)	29 (93.5%)	115 (77.2%)	*p* < 0.05
Lumbar spine (L3-L5)	2 (6.5%)	34 (22.8%)	*p* < 0.05
Old vertebral fracture	0.90 ± 0.18	0.59 ± 0.09	*p* < 0.05
Posterior wall injury (%)	48.4% (15/31)	32.9% (49/149)	N.S.
Intravertebral cleft	35.5% (11/31)	26.9% (40/149)	N.S.
Vacuum phenomenon in the adjacent intervertebral disc	25.8% (8/31)	11.4% (17/149)	N.S.
Cement volume (mL)	7.3 ± 0.27	7.3 ± 0.12	N.S.
Preoperative vertebral kyphosis angle	−10.7 ± 0.91	−9.5 ± 0.41	N.S.
Immediately postoperative vertebral kyphosis angle	−4.7 ± 0.59	−4.4 ± 0.27	N.S.
Final postoperative vertebral kyphosis angle	−7.9 ± 0.86	−6.6 ± 0.39	N.S.
Preoperative local kyphosis angle	−11.3 ± 2.54	−6.3 ± 1.16	N.S.
Immediately postoperative local kyphosis angle	−0.9 ± 3.05	−1.1 ± 1.39	N.S.
Final postoperative local kyphosis angle	−11.2 ± 2.74	−5.2 ± 1.23	*p* < 0.05
Preoperative vertebral wedge ratio (%)	69.5 ± 2.49	76.2 ± 1.13	*p* < 0.05
Immediately postoperative vertebral wedge ratio (%)	86.5 ± 1.58	88.6 ± 0.72	N.S.
Final postoperative vertebral wedge ratio (%)	78.7 ± 2.33	82.1 ± 1.05	N.S.

AVF, adjacent vertebral fracture; YAM, young adult mean. N.S.: not significant.

**Table 2:** Distribution of AVF by Vertebral Level.

	Upper Vertebral	Lower Vertebral	*p*-Value
AVF levels	22/31 (71.0%)	9/31 (29.0%)	*p* < 0.05

AVF, adjacent vertebral fracture.

**Table 3:** Mean HU Values at ROI A–F.

	AVF Group	Non-AVF Group	*p*-Value
A	53.6 ± 68.9	94.0 ± 79.5	*p* < 0.05
B	57.3 ± 72.3	76.7 ± 81.0	*p* < 0.05
C	62.9 ± 67.2	86.2 ± 76.2	*p* < 0.05
D	50.7 ± 69.7	94.3 ± 79.6	*p* < 0.05
E	46.8 ± 71.2	72.2 ± 85.7	*p* < 0.05
F	47.9 ± 68.1	83.0 ± 83.4	*p* < 0.05

AVF, adjacent vertebral fracture; HU, Hounsfield unit; ROI, region of interest.

**Table 4:** Mean HU Values of Upper and Lower Adjacent Vertebrae.

	AVF Group (*n* = 31)	Non-AVF Group (*n* = 149)	*p*-Value
Upper vertebral CT values (A + B + C/3)	61.1 ± 6.03	84.7 ± 2.75	*p* < 0.05
Lower vertebral CT values (D + E + F/3)	51.5 ± 8.44	81.0 ± 3.85	*p* < 0.05

AVF, adjacent vertebral fracture; CT, computed tomography; HU, Hounsfield unit.

**Table 5:** Multivariable Logistic Regression Analysis for AVF Risk Factors.

Risk Factor	Odds Ratio (95% Confidence Interval)	*p*-Value
Old vertebral fracture	1.12 (0.73–1.64)	0.571
Final postoperative local kyphosis angle	0.97 (0.93–1.01)	0.188
Preoperative vertebral wedge ratio	0.97 (0.94–1.01)	0.250
Upper vertebral CT values (A + B + C/3)	0.97 (0.95–0.99)	0.043
Lower vertebral CT values (D + E + F/3)	0.98 (0.96–0.99)	0.048

AVF, adjacent vertebral fracture; CT, computed tomography

## Data Availability

Individual data cannot be disclosed because of privacy and ethical constraints.

## Abbreviations

The following abbreviations are used in this manuscript:

ADL	Activities of Daily Living
AVF	Adjacent Vertebral Fracture
AUC	Area Under the Curve
BKP	Balloon Kyphoplasty
BMD	Bone Mineral Density
CI	Confidence Interval
CT	Computed Tomography
DISH	Diffuse Idiopathic Skeletal Hyperostosis
DEXA	Dual-Energy X-ray Absorptiometry
HU	Hounsfield Unit
OR	Odds Ratio
OVF	Osteoporotic Vertebral Fracture
PVP	Percutaneous Vertebroplasty
ROC	Receiver Operating Characteristic
ROI	Region of Interest
SD	Standard Deviation
YAM	Young Adult Mean
